# Scored minor criteria for severe community-acquired pneumonia predicted better

**DOI:** 10.1186/s12931-019-0991-4

**Published:** 2019-01-31

**Authors:** Qi Guo, Wei-dong Song, Hai-yan Li, Yi-ping Zhou, Ming Li, Xiao-ke Chen, Hui Liu, Hong-lin Peng, Hai-qiong Yu, Xia Chen, Nian Liu, Zhong-dong Lü, Li-hua Liang, Qing-zhou Zhao, Mei Jiang

**Affiliations:** 1 Department of Respiratory Medicine, Shenzhen Hospital, Peking University, Lianhua road No. 1120, Shenzhen, 518036 Guangdong China; 2 Department of Respiratory Medicine, The Eighth Affiliated Hospital (Shenzhen Futian), Sun Yat-sen University, Shenzhen, 518033 Guangdong China; 3 Medical Department, The Eighth Affiliated Hospital (Shenzhen Futian), Sun Yat-sen University, Shenzhen, 518033 Guangdong China; 4 Department of Radiology, The Eighth Affiliated Hospital (Shenzhen Futian), Sun Yat-sen University, Shenzhen, 518033 Guangdong China; 5 Guangzhou Institute of Respiratory Diseases (State Key Laboratory of Respiratory Diseases), First Affiliated Hospital, Guangzhou Medical University, Guangzhou, 510120 Guangdong China

**Keywords:** Community-acquired pneumonia, Minor criteria, Score, Mortality, Severity

## Abstract

**Background:**

Infectious Disease Society of America/American Thoracic Society (IDSA/ATS) minor criteria for severe community-acquired pneumonia (CAP) are of unequal weight in predicting mortality, but the major problem associated with IDSA/ATS minor criteria might be a lack of consideration of weight in prediction in clinical practice. Would awarding different points to the presences of the minor criteria improve the accuracy of the scoring system? It is warranted to explore this intriguing hypothesis.

**Methods:**

A total of 1230 CAP patients were recruited to a retrospective cohort study. This was tested against a prospective two-center cohort of 1749 adults with CAP. 2 points were assigned for the presence of PaO_2_/FiO_2_ ≤ 250 mmHg, confusion, or uremia on admission and 1 point for each of the others.

**Results:**

The mortality rates, and sequential organ failure assessment (SOFA) and pneumonia severity index (PSI) scores increased significantly with the numbers of IDSA/ATS minor criteria present and minor criteria scores. The correlations of the minor criteria scores with the mortality rates were higher than those of the numbers of IDSA/ATS minor criteria present. As were the correlations of the minor criteria scores with SOFA and PSI scores, compared with the numbers of IDSA/ATS minor criteria present. The pattern of sensitivity, specificity, positive predictive value, and Youden’s index of scored minor criteria of ≥2 scores or the presence of 2 or more IDSA/ATS minor criteria for prediction of mortality was the best in the retrospective cohort, and the former was better than the latter. The validation cohort confirmed a similar pattern. The area under the receiver operating characteristic curve of scored minor criteria was higher than that of IDSA/ATS minor criteria in the retrospective cohort, implying higher accuracy of scored version for predicting mortality. The validation cohort confirmed a similar paradigm.

**Conclusions:**

Scored minor criteria orchestrated improvements in predicting mortality and severity in patients with CAP, and scored minor criteria of ≥2 scores or the presence of 2 or more IDSA/ATS minor criteria might be more valuable cut-off value for severe CAP, which might have implications for more accurate clinical triage decisions.

## Background

Community-acquired pneumonia (CAP) is the most common cause of mortality from infectious diseases and a big burden to finite hospital and intensive care unit (ICU) resources [1]. Significant improvements in treatment of CAP have been emerging, but mortality remains unacceptably high [2, 3]. In 2007, the Infectious Disease Society of America and the American Thoracic Society (IDSA/ATS) designed minor criteria with the aim to guide ICU admission, not to predict mortality [2]. We [4] and Sibila et al. [5] have reported that some of these criteria might be predictors of mortality, while others not. The minor criteria are of unequal weight in predicting mortality and some of these criteria could be removed to orchestrate a simplified version [4, 6–10]. Therefore, the major problem associated with IDSA/ATS minor criteria might be a lack of consideration of weight in prediction in clinical practice.

We found that mortality among patients with severe CAP depended on combinations of IDSA/ATS minor criteria and the combination of arterial oxygen pressure/fraction inspired oxygen (PaO_2_/FiO_2_) ≤ 250 mmHg, confusion and uremia predicted higher mortality [11]. PaO_2_/FiO_2_ ≤ 250 mmHg, confusion and uremia had the strongest association with mortality based on what we [4, 11], Brown et al., [6] Liapikou et al., [7] and Phua et al. [8] reported. Consequently, would awarding 2 points to the presence of PaO_2_/FiO_2_ ≤ 250 mmHg, confusion or uremia improve the accuracy of the scoring system? The more accurate the scoring system, the higher the patient survival. Hence, it is worthwhile to explore this intriguing hypothesis.

Two cohort studies were conducted to derive and validate a scored minor criteria .

## Materials and methods

### Design and setting

A retrospective cohort study of 1245 adult patients with CAP was conducted at the Department of Respiratory Medicine in a Chinese affiliated tertiary hospital of a medical university from 2005 to 2009. We performed a prospective two-centre cohort study of 1779 consecutive adult patients with CAP between 2010 and 2014 at the Departments of Respiratory Medicine in two Chinese affiliated tertiary hospitals of two medical universities, one of which was the same hospital in the retrospective cohort study.

### Criteria for enrollment

CAP was defined as an acute infection of the pulmonary parenchyma associated with an acute infiltrate on the chest radiograph with two or more symptoms including fever (> 38 °C), hypothermia (< 36 °C), rigors, sweats, new cough or change in color of respiratory secretions, chest discomfort or dyspnoea [8]. Patients who were younger than 18 years, who had been hospitalized during the 28 days preceding the study, who had severe immunosuppression, active tuberculosis, or end-stage diseases, who had a written “do not resuscitate” order, or whose baseline consciousness was unclear, which was not derived from pneumonia, were excluded.

### Clinical management

Patients with CAP were admitted and attended by respiratory physicians based on the ATS guidelines [2, 12] and the Surviving Sepsis Campaign guidelines [13, 14]. The initial antibiotic regimens were consistent with the guidelines on the management of CAP, in addition to subsequently cultured pathogens. Therefore, all patients were regarded as receiving adequate antibiotics and were discharged home when they reached clinical stability and became afebrile.

### Score assigned for each of IDSA/ATS minor criteria

On the basis of the weight of IDSA/ATS minor criteria for severe CAP in predicting mortality, two points were assigned for the presence of PaO_2_/FiO_2_ ≤ 250 mmHg, confusion, or uremia on admission to the hospitals and one point for each of the others in scored minor criteria scoring system (Table 1).

**Table 1:** The minor criteria scoring systems

Weight Variable	IDSA/ATS minor criteria	Scored minor criteria
Respiratory rate ≥ 30 breaths/min	1	1
PaO_2_/FiO_2_ ≤ 250 mmHg	1	2
Multilobar infiltrates	1	1
Confusion	1	2
Uremia	1	2
Leukopenia	1	1
Thrombocytopenia	1	1
Hypothermia	1	1
Hypotension	1	1
Total	9 variables	12 scores

NOTE: IDSA/ATS: The Infectious Disease Society of America and the American Thoracic Society. PaO_2_/FiO_2_: Arterial oxygen pressure/fraction inspired oxygen

#### Outcome

The main outcome measure was 28-day mortality. Secondary outcomes incorporated sequential organ failure assessment (SOFA) and pneumonia severity index (PSI) scores at 72 h after commencing therapy.

#### Data collection

**Fig. 1:**
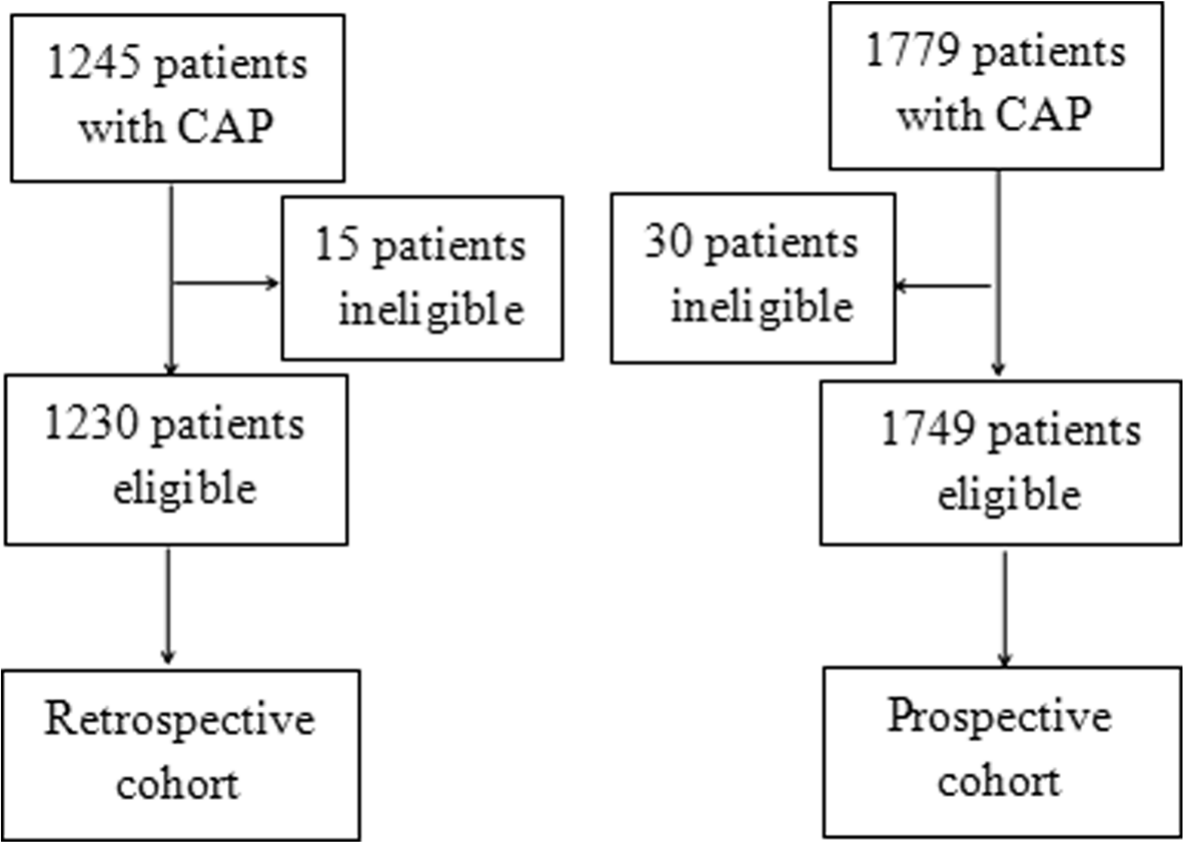
Study flow chart

A total of 1245 patients were enrolled consecutively and 15 cases were excluded from the retrospective cohort due to exclusion criteria (2 patients younger than 18 years, 1 patient hospitalized during the 28 days preceding the study, 6 patients with severe immunosuppression, 2 patients with active tuberculosis, 2 patients with end-stage diseases, 1 patient with a written “do not resuscitate” order, and 1 patient with unclear baseline consciousness). 30 cases were excluded from 1779 consecutive patients in the validation cohort (3 patients younger than 18 years, 4 patients hospitalized during the 28 days preceding the study, 9 patients with severe immunosuppression, 2 patients with active tuberculosis, 5 patients with end-stage diseases, 4 patients with a written “do not resuscitate” order, and 3 patients with unclear baseline consciousness) (Fig. 1). All the patients had chest radiographys and computer tomography (CT) scans. The frontal and lateral chest radiographic findings and CT scan images were classified independently by two senior radiologists (LH Liang and QZ Zhao). Clinical and diagnostic data, and radiological features were collected. Missing values, e.g. PaO_2_/FiO_2_ ≤ 250 mmHg, were assumed to be normal. CURB-65 (Confusion, Urea > 7 mmol·L^− 1^, Respiratory rate ≥ 30·min^− 1^, low Blood pressure, and age ≥ 65 yrs) scores on admission were calculated. SOFA and PSI scores at 72 h after start of therapy were calculated. Laboratory variables were measured by the hospital clinical laboratories. The statistician was blinded to the study.

#### Statistical analysis

All statistical analyses were performed with Statistical Package for the Social Science for Windows version 16.0 (SPSS, Chicago, IL, USA) and MedCalc version 17.9.2 (Mariakerke, Belgium). Categorical variables and continuous variables were reported as the percentages and the mean ± standard deviation (SD), respectively. Chi-Square test, one-way ANOVA, and Spearman rank correlation were employed. The receiver operating characteristic (ROC) curves were created and the areas under the curves (AUCs) were calculated to illustrate and compare the accuracy of the indices. The sensitivities, specificities, positive predictive values (PPVs), negative predictive values (NPVs), and Youden’s indices were also calculated. A *p* value of < 0.05 was considered statistically significant.

## Results

### Baseline characteristics of study cohorts

The baseline characteristics of the patients were summarized in Table 2. The IDSA/ATS minor criteria present in the prospective cohort were observed more frequently than those in the retrospective cohort and consequently more severely ill patients were recruited to the prospective cohort. The statuses of comorbidities, alcohol abuse, and smoking in the two cohort were similar.

**Table 2:** Baseline characteristics of study cohorts (Mean ± SD)

Characteristic	Retrospective cohort (*n* = 1230)	Validation cohort (*n* = 1749)
Age (yrs)	47.5 ± 22.2	50.1 ± 22.7
Male sex (%)	49.3	46.5
Hospital Length of stay (days)	10.1 ± 6.4	11.2 ± 7.5
Age ≥ 65 yrs. (%) (No.)	27.3 (336)	32.3 (565)
Comorbidities (%) (No.)		
Hypertension	29.3 (360)	31.1 (544)
Coronary heart disease	8.5 (105)	9.4 (164)
Heart failure	3.1 (38)	4.2 (73)
Chronic obstructive pulmonary disease	5.7 (70)	6.1 (107)
Diabetes mellitus	7.4 (91)	6.2 (108)
Chronic renal insufficiency	3.8 (47)	4.9 (86)
Liver disease	4.2 (52)	5.3 (93)
Nervous system disease	3.9 (48)	4.5 (79)
Tumour	6.8 (84)	7.7 (135)
Alcohol abuse (%) (No.)	3.2 (39)	2.9 (51)
Smoking (%) (No.)	26.3 (323)	27.8 (486)
Respiratory rate ≥ 30 breaths/min (%) (No.)	2.4 (30)	10.9 (191)
PaO_2_/FiO_2_ ≤ 250 mmHg (%) (No.)	3.1 (38)	15.1 (264)
Multilobar infiltrates (%) (No.)	27.2 (334)	39.8 (696)
Confusion (%) (No.)	1.8 (22)	6.4 (112)
Uremia (%) (No.)	6.3 (78)	17.5 (306)
Leukopenia (%) (No.)	5.4 (66)	7.8 (136)
Thrombocytopenia (%) (No.)	2.3 (28)	5.3 (93)
Hypothermia (%) (No.)	4.2 (52)	6.5 (114)
Hypotension (%) (No.)	14.3 (176)	21.0 (367)

NOTE: PaO_2_/FiO_2_: Arterial oxygen pressure/fraction inspired oxygen

### Associations with 28-day mortality

The 28-day mortalities were 1.3 and 4.5% in the retrospective and prospective cohorts, respectively. The mortality rates in the retrospective cohort were positively associated with the numbers of IDSA/ATS minor criteria present and minor criteria scores (*x*^*2*^, *p*. 108.434, < 0.001; 153.268, < 0.001; respectively. Table 3). The validation cohort confirmed a similar paradigm (*x*^*2*^, *p*. 179.674, < 0.001; 461.356, < 0.001; respectively. Table 3). The correlation of the minor criteria scores with the mortality rates was higher than that of the numbers of IDSA/ATS minor criteria present in the retrospective cohort (Rank correlation coefficient value, *p.* 0.434, < 0.001; 0.300, < 0.001; respectively). The prospective cohort confirmed a similar pattern (Rank correlation coefficient value, *p.* 0.504, < 0.001; 0.353, < 0.001; respectively).

**Table 3:** Relationship between number of adverse features and risk of mortality

Features	No. Present or score	Retrospective cohort (*n* = 1230)		Validation cohort (*n* = 1749)	
		Total	Died (%)	Total	Died (%)
IDSA/ATS minor criteria					
	0	654	2 (0.3)	714	2 (0.3)
	1	402	4 (1.0)	377	6 (1.6)
	2	120	4 (3.3)	210	14 (6.7)
	3	38	4 (10.5)	261	28 (10.7)
	4	12	0 (0)	133	11 (8.3)
	5	4	2 (50.0)	54	18 (33.3)
Scored minor criteria					
	0	654	2 (0.3)	714	2 (0.3)
	1	388	2 (0.5)	354	4 (1.1)
	2	96	2 (2.1)	95	4 (4.2)
	3	49	4 (8.2)	113	7 (6.2)
	4	21	2 (9.5)	231	15 (6.5)
	5	15	2 (13.3)	141	19 (13.5)
	6	4	1 (25.0)	36	7 (19.4)
	7	3	1 (33.3)	11	3 (27.3)
	8			54	18 (33.3)

NOTE: IDSA/ATS: The Infectious Disease Society of America and the American Thoracic Society

### SOFA and PSI scores according to the predictive findings

SOFA and PSI scores increased significantly with the numbers of IDSA/ATS minor criteria present in the two cohorts (Table 4), and all the differences between the groups were significant (*p* <  0.001). As did SOFA and PSI scores with the minor criteria scores (Table 4). The numbers of IDSA/ATS minor criteria present were positively associated with SOFA and PSI scores in the two cohorts (Table 4). The associations of the minor criteria scores with SOFA and PSI scores confirmed similar paradigms, and the rank correlation coefficient values were higher than the corresponding ones, compared with the numbers of IDSA/ATS minor criteria present.

**Table 4:** SOFA and PSI scores according to the number of minor criteria present and minor criteria score (Mean ± SD)

NO. of minor criteria/Minor criteria scores	Retrospective cohort (*n* = 1230)		Prospective cohort (*n* = 1749)	
	SOFA score	PSI score	SOFA score	PSI score
None minor criteria	0.31 ± 0.64	28.63 ± 14.49	0.30 ± 0.60	26.51 ± 15.38
One minor criteria	0.64 ± 1.10	41.35 ± 22.07	0.69 ± 0.81	50.62 ± 21.87
Two minor criteria	1.32 ± 1.43	67.08 ± 16.25	1.74 ± 1.51	77.25 ± 19.33
Three minor criteria	3.58 ± 1.98	89.83 ± 21.60	3.26 ± 1.90	109.27 ± 20.14
Four minor criteria	3.00 ± 1.04	117.53 ± 16.48	4.35 ± 1.92	128.49 ± 17.45
Five minor criteria	6.50 ± 1.73	139.07 ± 12.36	6.99 ± 1.32	149.53 ± 13.92
*F* value	138.004	159.473	174.697	162.536
*p* value	< 0.001	< 0.001	< 0.001	< 0.001
Rank correlation coefficient (*r*_s_) value	0.354	0.601	0.765	0.637
*p* value	< 0.001	< 0.001	< 0.001	< 0.001
Zero score	0.31 ± 0.64	28.63 ± 14.49	0.30 ± 0.60	26.51 ± 15.38
One score	0.52 ± 0.88	39.72 ± 19.84	0.41 ± 0.67	35.18 ± 17.42
Two scores	1.24 ± 1.54	64.08 ± 15.38	1.28 ± 1.50	70.32 ± 14.59
Three scores	3.14 ± 1.62	85.29 ± 19.17	2.06 ± 1.62	83.17 ± 18.25
Four scores	4.14 ± 1.42	109.71 ± 18.25	3.12 ± 1.48	99.38 ± 16.42
Five scores	5.37 ± 1.13	125.38 ± 13.79	4.03 ± 1.32	118.46 ± 13.22
Six scores	6.21 ± 1.62	138.94 ± 14.21	5.38 ± 1.53	132.58 ± 15.71
Seven scores	7.67 ± 1.10	159.34 ± 16.57	6.87 ± 1.49	150.16 ± 14.28
Eight scores			7.70 ± 1.07	167.95 ± 15.83
*F* value	158.356	167.385	187.216	194.576
*p* value	< 0.001	< 0.001	< 0.001	< 0.001
Rank correlation coefficient (*r*_s_) value	0.617	0.725	0.821	0.859
*p* value	< 0.001	< 0.001	< 0.001	< 0.001

NOTE: SOFA: Sequential organ failure assessment. PSI: Pneumonia severity index

### Comparisons of the scoring systems for predicting 28-day mortality

The sensitivities, specificities, and predictive values of the different scoring systems for predicting mortality were shown in Table 5. The pattern of sensitivity, specificity, PPV, and Youden’s index of scored minor criteria of ≥2 scores or the presence of 2 or more IDSA/ATS minor criteria for prediction of mortality was the best in the retrospective cohort. The pattern of sensitivity, specificity, PPV, and Youden’s index of scored minor criteria of ≥3 or ≥ 2 scores for prediction of mortality was better than that of the presence of ≥3 or ≥ 2 IDSA/ATS minor criteria in the retrospective cohort, respectively. High values of corresponding indices were confirmed in the prospective cohort. Therefore, scored minor criteria of ≥2 scores or the presence of 2 or more IDSA/ATS minor criteria might be more valuable cut-off value for severe CAP.

**Table 5:** Test characteristics of rules with different prediction scores for mortality in the retrospective and prospective sets of patients hospitalized with CAP

Rule	No. Present or score	Sensitivity (%)	Specificity (%)	PPV (%)	NPV (%)	Youden’s index
Retrospective cohort (*n* = 1230)						
IDSA/ATS minor criteria						
	≥ 0	100	0	1.3	0	0
	≥ 1	87.5	53.7	2.4	99.7	0.41
	≥ 2	62.5	86.5	5.7	99.4	0.49
	≥ 3	37.5	96	11.1	99.1	0.34
	≥ 4	12.5	98.8	12.5	98.8	0.11
	≥ 5	12.5	99.8	50	98.9	0.12
Scored minor criteria						
	≥ 0	100	0	1.3	0	0
	≥ 1	87.5	53.7	2.4	99.7	0.41
	≥ 2	75	85.5	6.4	99.6	0.61
	≥ 3	62.5	93.2	10.9	99.5	0.56
	≥ 4	37.5	97	14	99.2	0.35
	≥ 5	25	98.5	18.2	99	0.24
	≥ 6	6.3	99.5	14.3	98.8	0.06
	≥ 7	6.3	99.8	33.3	98.8	0.06
Prospective cohort (n = 1749)						
IDSA/ATS minor criteria						
	≥ 0	100	0	4.5	0	0
	≥ 1	97.5	42.6	7.4	99.7	0.40
	≥ 2	89.9	64.9	10.8	99.3	0.55
	≥ 3	72.2	76.6	12.7	98.3	0.49
	≥ 4	36.7	90.5	15.5	96.8	0.27
	≥ 5	22.8	97.8	33.3	96.4	0.21
Scored minor criteria						
	≥ 0	100	0	4.5	0	0
	≥ 1	97.5	42.6	7.4	99.7	0.40
	≥ 2	92.4	63.6	10.7	99.4	0.56
	≥ 3	87.3	69.0	11.8	99.1	0.56
	≥ 4	78.5	75.4	13.1	98.7	0.54
	≥ 5	59.5	88.3	19.4	97.9	0.48
	≥ 6	35.4	95.6	27.7	96.9	0.31
	≥ 7	26.6	95.6	32.3	96.6	0.22
	≥ 8	22.8	97.8	33.3	96.4	0.21

NOTE: CAP: Community-acquired pneumonia. PPV: Positive predictive value. NPV: Negative predictive value. IDSA/ATS: The Infectious Disease Society of America and the American Thoracic Society

**Fig. 2:**
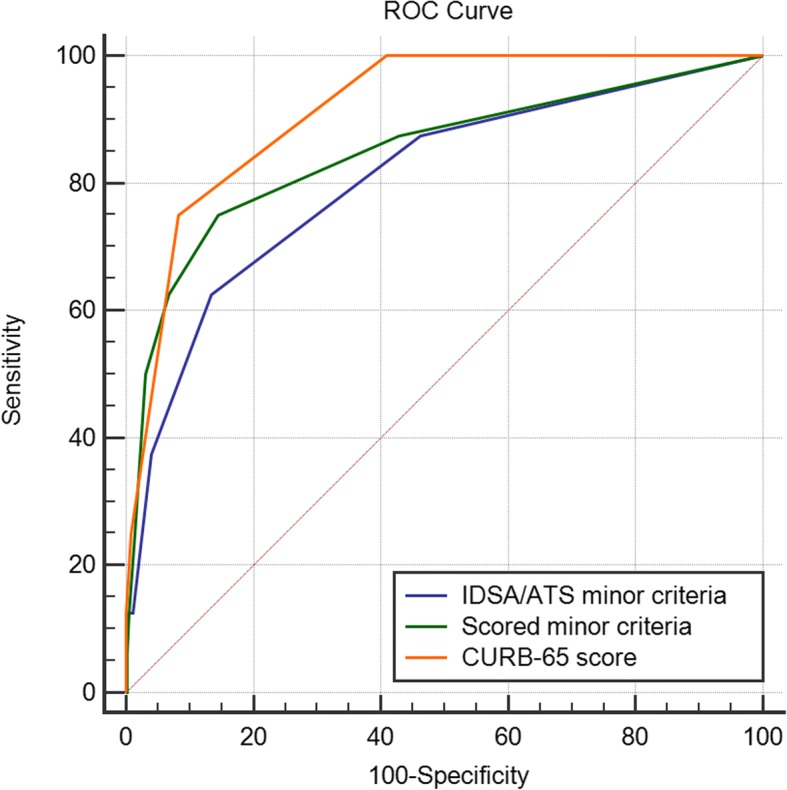
ROC curves for mortality prediction in the retrospective cohort

**Fig. 3:**
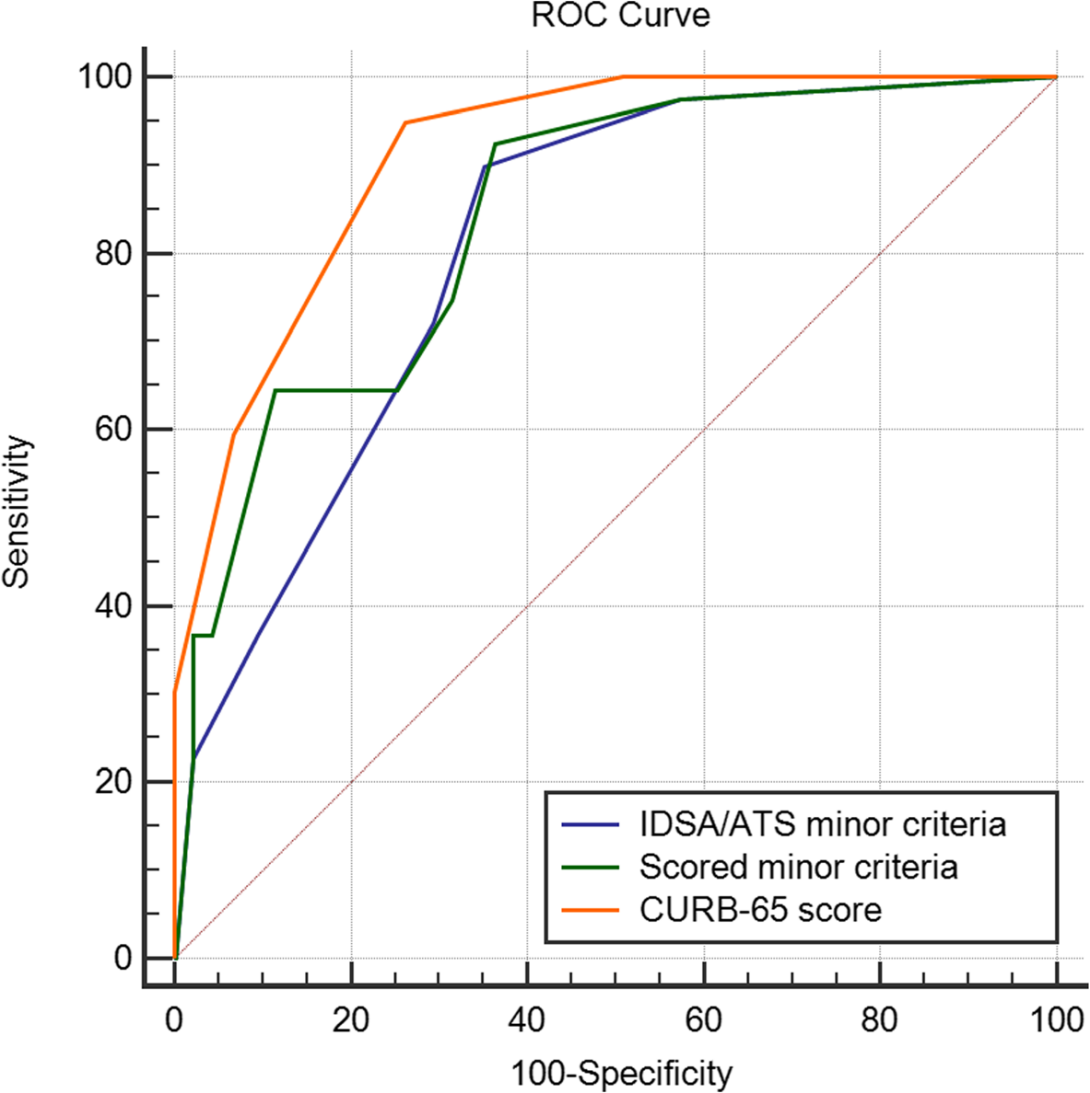
ROC curves for mortality prediction in the validation cohort

The ROC curves for the two minor criteria scoring systems and CURB-65 score in the two study populations illustrated the differences in accuracy of mortality prediction (Tables 6 and 7, and Figs. 2 and 3). Scored minor criteria was performed worse than CURB-65 score in the prospective cohort, but it was performed better in the two cohorts, compared with IDSA/ATS minor criteria.

**Table 6:** AUC values for different scoring systems

Feature	Retrospective cohort (*n* = 1230)			Validation cohort (*n* = 1749)		
	AUC value	Standard error	95% CI	AUC value	Standard error	95% CI
IDSA/ATS minor criteria	0.805	0.0599	0.782–0.827	0.808	0.0197	0.789–0.826
Scored minor criteria	0.848	0.0596	0.827–0.868	0.840	0.0208	0.822–0.857
CURB-65 score	0.915	0.0249	0.898–0.930	0.912	0.0118	0.898–0.925

NOTE: *AUC* The area under the receiver operating characteristic curve. *CI* Confidence interval. *IDSA/ATS* The Infectious Disease Society of America and the American Thoracic Society. *CURB-65* Confusion, Urea > 7 mmol·L^−1^, Respiratory rate ≥ 30·min^−1^, low Blood pressure, and age ≥ 65 yrs

**Table 7:** Comparison of AUC values between the scoring systems

Feature	Retrospective cohort (*n* = 1230)			Validation cohort (*n* = 1749)		
	Difference	z statistic	*p* value	Difference	z statistic	*p* value
IDSA/ATS ~ Scored	0.0426	2.635	0.0084	0.0316	4.295	< 0.0001
IDSA/ATS ~CURB-65	0.110	2.609	0.0091	0.104	4.742	< 0.0001
Scored ~ CURB-65	0.0672	1.672	0.0944	0.0722	4.094	< 0.0001

NOTE: *AUC* The area under the receiver operating characteristic curve. *IDSA/ATS* The Infectious Disease Society of America and the American Thoracic Society. *CURB-65* Confusion, Urea > 7 mmol·L^−1^, Respiratory rate ≥ 30·min^− 1^, low Blood pressure, and age ≥ 65 yrs

## Discussion

The main findings of the current study comprise the following: The mortality rates, and SOFA and PSI scores increased significantly with the numbers of IDSA/ATS minor criteria present and minor criteria scores. The correlations of the minor criteria scores with the mortality rates were higher than those of the numbers of IDSA/ATS minor criteria present. As were the correlations of the minor criteria scores with SOFA and PSI scores, compared with the numbers of IDSA/ATS minor criteria present. The pattern of sensitivity, specificity, PPV, and Youden’s index of scored minor criteria of ≥2 scores or the presence of 2 or more IDSA/ATS minor criteria for prediction of mortality was the best in the two cohorts. Scored minor criteria of ≥2 scores or the presence of 2 or more IDSA/ATS minor criteria might be more valuable cut-off value for severe CAP. The pattern of sensitivity, specificity, PPV, and Youden’s index of scored minor criteria of ≥3 or ≥ 2 scores for prediction of mortality was better than that of the presence of ≥3 or ≥ 2 IDSA/ATS minor criteria, respectively. The higher accuracies of scored minor criteria for predicting mortality in the two cohorts were illustrated by the higher AUC values, compared with IDSA/ATS minor criteria.

It may be necessary to perform local recalibration of the score were the population of patients to which the score is being applied significantly different from the original derivation [15]. Patients with 3 or more IDSA/ATS minor criteria are at high risk of death, which was derived from target populations with high mortality [2]. The patients meeting 3 scores/variables also presented higher mortalities than those with 2 scores/variables in the current study. However, the pattern of sensitivity, specificity, PPV, and Youden’s index of scored minor criteria of ≥2 scores or the presence of 2 or more IDSA/ATS minor criteria for prediction of mortality was the best in the current two cohorts. Low mortality rate might be envisaged to interpret this seemingly paradoxical phenomenon, as we reported previously [16]. Based on low-mortality-rate, only a few patients met 3 or more scores/variables of the scoring system, incurring high false negative rate. On the contrary, had the cut-off value been reduced to 2 or more scores/variables, relatively more patients would have been better characterised as having severe CAP, ensuring lower false negative rate, with similar high NPV. Therefore, future prospective multicenter cohort studies are warranted to assess the generalizability of the current findings.

On the basis of the unequal weight and the reduction of valuable cut-off value, at least 2 scores were assigned to the patients with CAP fulfilling PaO_2_/FiO_2_ ≤ 250 mmHg, confusion, or uremia, who might be triaged directly to ICU if having adequate ICU beds or at any rate need for advance care. Our previous data analyses might provide evidence for the consideration of weight and the reduction. Interestingly, the patients with non-severe CAP fulfilling PaO_2_/FiO_2_ ≤ 250 mmHg, confusion, or uremia demonstrated unexpectedly higher mortality rates, and SOFA and PSI scores, compared with the patients with severe CAP, without the variables, and might have the priority for treatment and intensive care, suggesting that ICU admission might be warranted for CAP patients with one of the two major criteria or at least one of the three variables [17, 18]. Similarly, the recently developed quick sepsis-related organ failure assessment (qSOFA. Range, 0–3 points, with 1 point each for systolic hypotension [≤100 mmHg], tachypnea [≥22/min], or altered mentation) is a fast and easy screening method for patients with a suspected infection who are at increased risk of mortality outside of the ICU [19]. PaO_2_/FiO_2_ ≤ 250 mmHg and confusion are very similar to 2 of 3 qSOFA criteria. In a prospective validation study, patients with a suspected infection and a qSOFA score ≥ 2 had a mortality rate of 24% [20].

Loke’s systematic review and meta-analysis suggest that CURB-65 score performs well at identifying patients with pneumonia that have a low risk of death (average mortality 7.4%) [21], which might be envisaged to interpret the reason why scored minor criteria was performed worse in the current validation cohort, compared with CURB-65 score.

The validation cohort appears to be more severe than the retrospective cohort. Two facts might be envisaged to interpret this issue. A hospital with more beds was included in the prospective two-centre cohort study. The patients from the bigger hospital were more severe than those from the smaller one. The prospective cohort study was performed five years later, and more severe patients might be admitted. Therefore, the validation might seemingly become less robust. A bigger z statistic value and a smaller *p* value presented when considering AUC values between IDSA/ATS and scored minor criteria in the validation cohort, compared with those in the retrospective cohort (z, *p*. 4.295 vs 2.635, < 0.0001 vs 0.0084, respectively). Hence, scored minor criteria might also be suitable in high-mortality settings. Actually, the major problem in the application of the IDSA/ATS minor criteria might be of ignoring weight in prediction, which might underestimate some variables. On the contrary, the consideration of weight might embody the true features of the variables, which might not overestimate them in a population with more severe CAP.

The current study came from two low-mortality settings. The Chinese health care system and Chinese primary care system are both so different from those in other countries [22]. A typical Chinese inpatient CAP population is that the most are young patients admitted with mild CAP. This might seemingly be a limit for the generalizability of the results. Nevertheless, on the basis of the above-mentioned interpretation, the current findings might be feasible if applied in high-mortality settings. Furthermore, it looks paradoxical that in a non-severe group of patients a test that assesses for severity is used, but doubt on the seeming paradox should be cast.

It is a major challenge in the management of CAP to identify patients who might rapidly develop adverse medical outcomes among those without obvious reason for immediate ICU admission. The presences of 2007 IDSA/ATS minor criteria indicate that the corresponding organs and organ systems do not perform well. The kidney, lung and central nervous system play pivotal physiological roles in human life. Therefore, their dysfunctions are most strongly associated to mortality and severity. Hence, the assignment of different points elaborated the different weight of minor criteria in predicting mortality and severity, which might orchestrate improvements in prediction. There is not any study in the quantification of the weight of IDSA/ATS minor criteria in prediction in the NCBI database. It is not complicated and difficult to ensure full compliance with scored minor criteria scoring system in clinical practice. The consideration of weight in predicting mortality and severity and the decrease in valuable cut-off value might have implications for more accurate clinical triage decisions about where these patients should be treated at (ICU vs. non-ICU) and need for advance care, especially on patients with non-severe CAP and in low-mortality settings in the application of IDSA/ATS minor criteria, which may improve survival.

## Limitations

Several limitations of this study deserve comment. First, the prospective cohort was derived from two centers in a city, but not multicenter settings located in different cities in different countries. This may limit the generalizability of the results. Second, there were relatively small samples. Had the numbers been larger, perhaps the results might have been more robust. The frequencies of presences of some minor criteria were less than 10%, which might be able to underestimate the results. Oxygen therapy by oxygen mask or ventilator was employed was the oxygen saturation of a patient lower than 90%, but FiO_2_ is not so accurate by mask flow. The data about ICU utilization and ICU admission were not collected. Finally and most importantly, the length of the study period was long. The management of CAP had changed during this time period, so the groups might be less comparable.

## Conclusions

Scored minor criteria orchestrated improvements in predicting mortality and severity in patients with CAP, and scored minor criteria of ≥2 scores or the presence of 2 or more IDSA/ATS minor criteria might be more valuable cut-off value for severe CAP, which might have implications for more accurate clinical triage decisions.

## Abbreviations

AUC: The area under the receiver operating characteristic curve
CAP: Community-acquired pneumonia
CT: Computer tomography
CURB-65: Confusion, Urea > 7 mmol·L − 1, Respiratory rate ≥ 30·min − 1, low Blood pressure, and age ≥ 65 yrs.
ICU: Intensive care unit
IDSA/ATS: The Infectious Disease Society of America and the American Thoracic Society
NPV: Negative predictive value
PaO2/FiO2: Arterial oxygen pressure/fraction inspired oxygen
PPV: Positive predictive value
PSI: Pneumonia severity index
qSOFA: Quick sepsis-related organ failure assessmentl
ROC: The receiver operating characteristic
SD: The mean ± standard deviation
SOFA: Sequential organ failure assessment
