# Correction to: Scored minor criteria for severe community-acquired pneumonia predicted better

**DOI:** 10.1186/s12931-019-1122-y

**Published:** 2019-07-10

**Authors:** Qi Guo, Wei-dong Song, Hai-yan Li, Yi-ping Zhou, Ming Li, Xiao-ke Chen, Hui Liu, Hong-lin Peng, Hai-qiong Yu, Xia Chen, Nian Liu, Zhong-dong Lü, Li-hua Liang, Qing-zhou Zhao, Mei Jiang

**Affiliations:** 1 Department of Respiratory Medicine, Shenzhen Hospital, Peking University, Lianhua road No. 1120, Shenzhen, 518036 Guangdong China; 2 Department of Respiratory Medicine, The Eighth Affiliated Hospital (Shenzhen Futian), Sun Yat-sen University, Shenzhen, 518033 Guangdong China; 3 Medical Department, The Eighth Affiliated Hospital (Shenzhen Futian), Sun Yat-sen University, Shenzhen, 518033 Guangdong China; 4 Department of Radiology, The Eighth Affiliated Hospital (Shenzhen Futian), Sun Yat-sen University, Shenzhen, 518033 Guangdong China; 5 Guangzhou Institute of Respiratory Diseases (State Key Laboratory of Respiratory Diseases), First Affiliated Hospital, Guangzhou Medical University, Guangzhou, 510120 Guangdong China

**Correction to: Respir Res (2019) 20:22**

**https://doi.org/10.1186/s12931-019-0991-4**

Although the focus of our article in Respiratory Research [1] reports some novel data and has a different focus compared to our publications in The American Journal of the Medical Sciences [2] and Respiratory Medicine [3], we acknowledge that we have duplicated some text and used the same study populations within this article [1] as our previous articles [2, 3].

The database used for the article published in Respiratory Research [1] was the basic database, which came to the conclusion that the individual 2007 IDSA/ATS minor criteria for severe community-acquired pneumonia (CAP) were of unequal weight in predicting hospital mortality, SOFA scores, hospital length of stay, and costs. The retrospective database used for the article published in The American Journal of the Medical Sciences [2] was the same in Respiratory Medicine [3]. The databases used for the article published in Respiratory Research [1] were the same as those in The American Journal of the Medical Sciences [2]. The two articles were based on the same basic theory that 2007 IDSA/ATS minor criteria for severe CAP were of unequal weight in prediction. We introduced some interesting findings in The American Journal of the Medical Sciences [2], that the patients with non-severe CAP fulfilling the predictive findings most strongly associated to mortality, i.e. PaO2/FiO2 ≤ 250 mmHg, confusion, and uremia, demonstrated higher SOFA and PSI scores and mortality rates, and might have the priority for treatment and intensive care. Therefore, in Respiratory Research [1], we further proposed a scored minor criteria scoring system which orchestrated improvements in predicting mortality and severity in patients with CAP, and suggested that scored minor criteria of ≥2 scores or the presence of 2 or more IDSA/ATS minor criteria might be more valuable cut-off value for severe CAP.

We apologize for the inappropriate overlap between our three publications and our lack of transparency about the similarities between the three articles.
